# Hybrid CNN-embedding fusion with MFCC-SVM for speech emotion recognition: Random vs actor-wise evaluation on CREMA-D

**DOI:** 10.1371/journal.pone.0355238

**Published:** 2026-08-24

**Authors:** Parveen Kumari, Yogita Yashveer Raghav, Vimmi Kochher, Prashanth kumar katta, Abhilasha A, Prabhakar M, Jayanthiladevi A, Fikir Gizachew Belete

**Affiliations:** 1 Department of Computer Science and Engineering, Dronacharya College of Engineering, Gurugram, India; 2 Centre of Excellence-Cloud Computing, School of Engineering & Technology K.R. Mangalam University, Gurugram, India; 3 Department of Computer Science and Engineering, Guru Teg Bahadur Institute of Technology, Mayapuri, Delhi, India; 4 Department of restorative dental sciences, College of Dentistry, King Faisal University, Al-Ahsa, Saudi Arabia; 5 Department of Computer Science and Engineering, Jaipur Engineering College and Research Centre, Shri Ram ki Nangal, Jaipur, India; 6 Department of Computer Science and Engineering, Dayananda Sagar University, Bangalore, India; 7 Department of Computer Science and Engineering, BGSIT, Adichunchanagiri University, BG Nagara, Karnataka, India; 8 Department of Computer Science, College of Computing, Debre Berhan University, Debre Berhan, Ethiopia; Guangdong University of Petrochemical Technology, CHINA

## Abstract

Speech Emotion Recognition (SER) is an important component of human-centered intelligent systems, yet robust performance remains challenging when speaker identities differ between training and testing. This study presents a protocol-aware and reproducible comparison on the CREMA-D corpus using three pipelines: (i) a classical MFCC-based Support Vector Machine (SVM), (ii) a log-mel Convolutional Neural Network (CNN), and (iii) a lightweight hybrid model that concatenates handcrafted acoustic descriptors with CNN-derived embeddings and uses an SVM classifier. The methodological contribution is not a new standalone classifier; it is the controlled integration of identical preprocessing, random and actor-wise evaluation, five-seed robustness reporting, class-wise error analysis, and CPU-oriented deployment within one experimental framework. The Hybrid approach achieves the best overall performance, obtaining 62.03% ± 0.94% Macro-F1 on the random split and 58.09% ± 1.36% on the actor-wise split, outperforming MFCC+SVM (55.72% ± 0.95% and 52.05% ± 1.95%) and the log-mel CNN (50.20% ± 1.51% and 42.68% ± 1.84%). A Streamlit interface supports WAV upload, live prediction, and export of per-seed confusion matrices and summary figures. The results show that a controlled lightweight fusion framework can improve robustness while making the performance gap between speaker-overlapping and speaker-independent evaluation explicit.

## 1 Introduction

Speech Emotion Recognition (SER) aims to automatically infer a speaker’s emotional state from speech signals and has become an important component in affective computing, human–computer interaction, intelligent tutoring, call-center analytics, and assistive technologies. Despite long-standing progress, SER remains difficult in practical deployments because emotional expression varies across speakers, speaking styles, languages, and recording environments. Comprehensive reviews emphasize that feature choice, dataset properties, and evaluation protocols strongly influence reported performance, and that results obtained in controlled settings may not translate to real-world conditions [1,2].

One of the most fundamental challenges is generalization. Performance reporting in many SER studies commonly relies on random train/test splits, where utterances from the same speaker may appear in both training and test data. This setting can inflate scores, as models may exploit speaker identity cues or recording artifacts rather than emotion-related variations [1]. Speaker-independent evaluation (e.g., actor-wise splits) provides a more realistic estimate for unseen speakers, but it is often underreported or inconsistently applied, making fair comparison across methods difficult [2].

Another important gap concerns the way established SER components are assembled and evaluated. Although MFCCs, CNNs, SVMs, feature fusion, and deployment tools are individually well known, they are often studied under different preprocessing choices, split protocols, and reporting practices. Consequently, it remains difficult to determine whether an apparent gain comes from the model itself, speaker overlap, or experimental configuration. A controlled framework that compares classical, deep, and hybrid pipelines under identical preprocessing, both random and actor-wise splits, repeated seeds, and a reusable inference path provides methodological value even when the individual algorithms are established.

In response to these concerns, this paper presents a replicable comparative study on the CREMA-D dataset [3]. CREMA-D contains 7,442 emotionally expressive speech samples from 91 actors across six emotion categories (angry, disgust, fear, happy, neutral, sad), providing a strong basis for examining speaker variability. Three modeling strategies representing common SER paradigms are considered. The first is a classical baseline using handcrafted acoustic features (MFCC statistics along with complementary spectral/prosodic features) and a Support Vector Machine (SVM) classifier [4,5]. The second is a compact convolutional neural network (CNN) using log-mel spectrogram inputs, enabling representation learning directly from time-frequency patterns relevant to the task [6,7]. The third is a hybrid fusion model that combines handcrafted acoustic features with deep CNN embedding vectors and performs classification using an SVM. The motivation for this hybrid design is the complementary nature of the two representations: handcrafted descriptors can provide stable low-level cues, while CNN embeddings can capture discriminative time-frequency patterns learned from data [6].

To better assess generalization, results are reported for both a conventional random split and an actor-wise (speaker-independent) split, where training and test sets contain non-overlapping speakers. Accuracy, Unweighted Average Recall (UAR), and Macro-F1 are used for model comparison, while confusion matrices are used to analyze class-wise errors. Conventional regularization and augmentation methods are applied to improve robustness and reduce overfitting. Time and frequency masking, commonly referred to as SpecAugment, is used to encourage invariance in spectrogram representations [8]. Focal loss is employed to emphasize difficult-to-classify samples, and mixup-style interpolation is used to improve generalization [9,10]. Feature extraction and signal processing are implemented using standard audio tooling [11].

### 1.1 Research gap

The study addresses the following research gaps:

- **Protocol gap:** SER performance is often reported using random splits with possible speaker overlap, while speaker-independent evaluation is less consistently adopted, limiting conclusions about real-world generalization [1].
- **Fair comparison gap:** Classical baselines (e.g., MFCC+SVM) and compact deep models are frequently evaluated with different preprocessing and split protocols, making direct comparison unreliable [2].
- **Reproducibility and deployment gap:** Many studies do not report multi-seed robustness or provide an end-to-end pipeline that can be validated on unseen user-provided audio, limiting practical verification and transparency.
- Integration and interpretation gap: Established handcrafted, deep, and hybrid components are less commonly evaluated together under identical data handling, repeated seeds, and both speaker-overlapping and speaker-independent protocols, making the source and robustness of performance gains difficult to interpret.

### 1.2 Contributions

The main contributions of this work are as follows:

- A controlled benchmark is provided for MFCC+SVM, a log-mel CNN, and a lightweight hybrid model under the same waveform standardization, label space, metrics, and software pipeline.
- A simple, CPU-compatible fusion implementation combines pooled handcrafted acoustic descriptors with penultimate-layer CNN embeddings and evaluates whether the complementary representations provide consistent gains.
- Random and actor-wise (speaker-independent) protocols are reported side by side to quantify the effect of speaker overlap on apparent performance.
- Five-seed mean ± standard deviation reporting and raw plus normalized confusion matrices are used to expose variability and class-wise error patterns rather than relying on a single headline score.
- A CPU-oriented Streamlit interface supports WAV upload, live prediction, and export of evaluation artifacts using the same preprocessing assumptions as the experiments.

### 1.3 Nature of novelty

The contribution of this study is methodological and practical rather than the invention of a new fundamental classifier. MFCCs, CNNs, SVMs, feature concatenation, and Streamlit are established techniques. The novelty lies in evaluating them as one controlled, protocol-aware framework in which the same standardized waveform feeds classical, deep, and hybrid branches; random and actor-wise splits are reported side by side; all results are repeated over five seeds; class-wise errors are retained as raw and normalized confusion matrices; and the trained artifacts are exposed through a CPU-oriented interface that uses the same preprocessing assumptions. This integrated design makes speaker leakage, robustness, and deployment reproducibility directly observable rather than hidden behind a single performance value.

Accordingly, the paper does not claim architectural novelty for the individual components. It contributes an experimentally transparent and lightweight reference design for interpreting representation fusion and speaker-independent generalization on CREMA-D.

**Fig 1:**
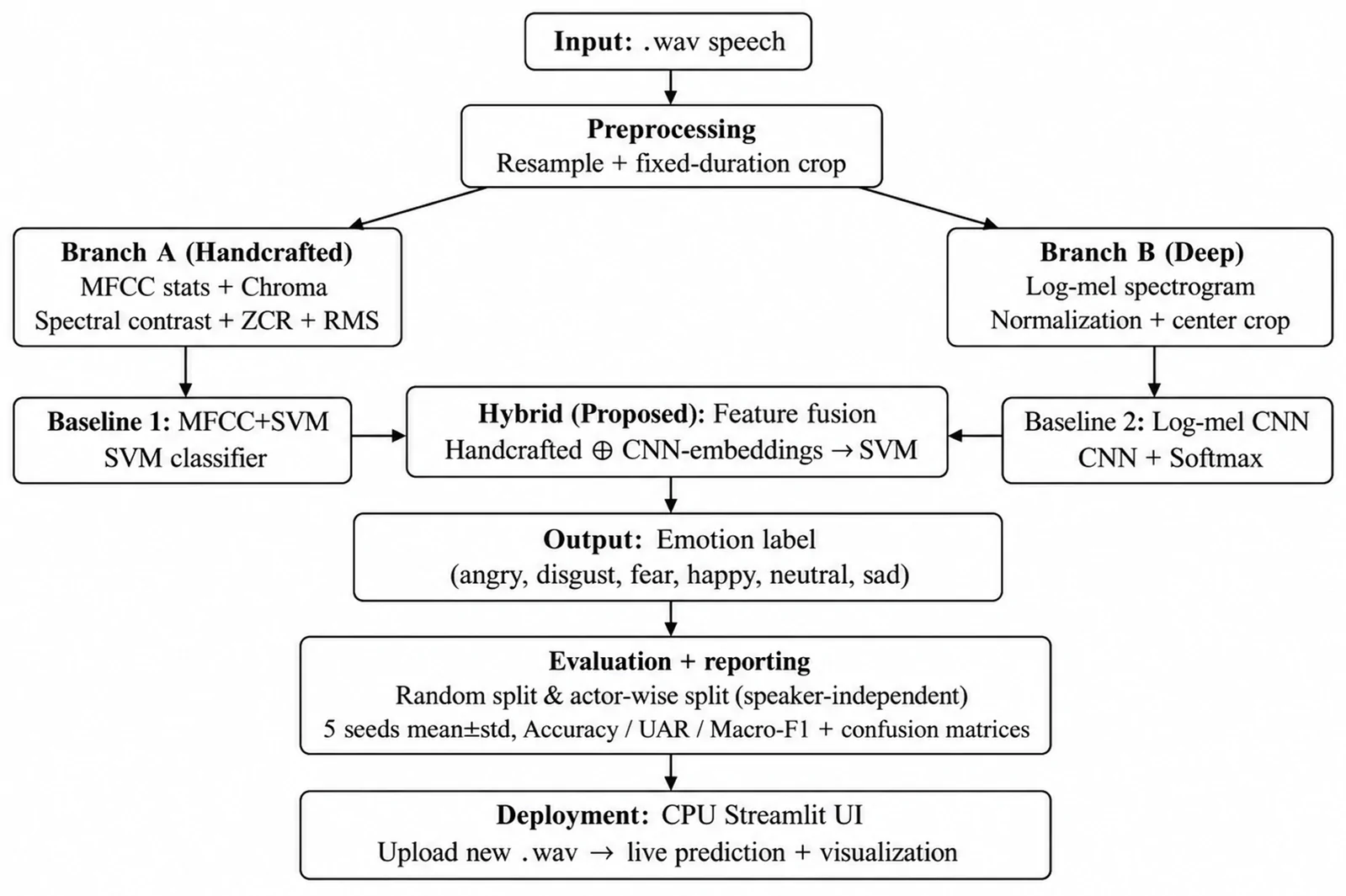
SER framework integrating MFCC–SVM, log-mel CNN, hybrid fusion, protocol-aware evaluation, and CPU inference.

**Fig 2:**
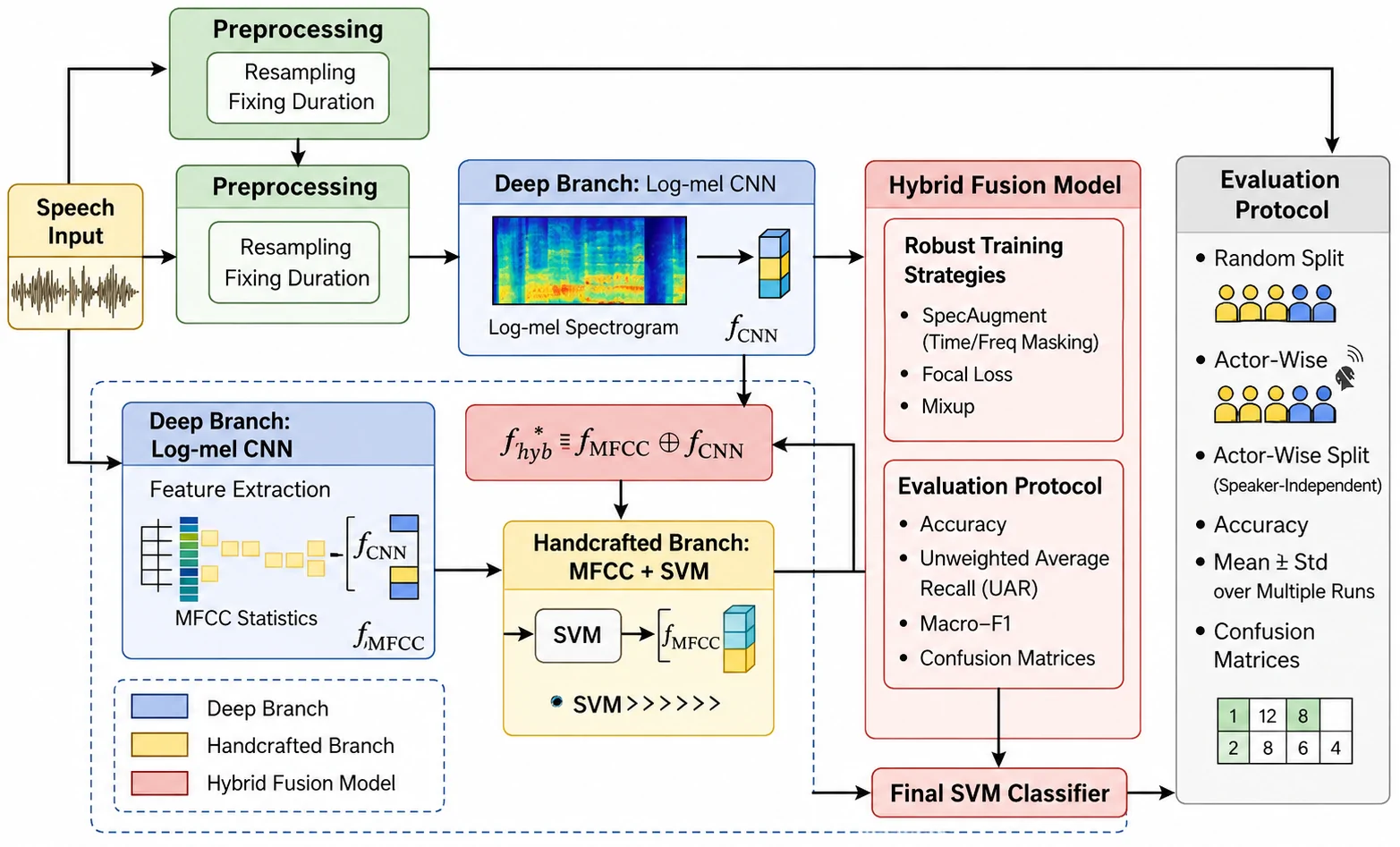
Proposed SER framework with preprocessing, MFCC–SVM, log-mel CNN, hybrid fusion, and protocol-aware evaluation.

As shown in Fig 1, the SER workflow uses two parallel representations derived from the same preprocessed waveform: handcrafted acoustic statistics and a log-mel spectrogram. The detailed protocol-aware processing path, including the three comparable pipelines and evaluation flow, is summarized in Fig 2.

## 2 Related work

Speech Emotion Recognition (SER) has developed out of the initial acoustic pipeline handcrafted pipelines to deep representation learning, attention-based architectures, transformer-like encoders, as well as, more recently, self-supervised speech models. With this development, two issues have remained to influence the field. First, reported performance is very sensitive to the characteristics of data and the evaluation protocol. Second, it can be challenging to meaningfully compare studies due to significant differences in preprocessing pipelines, feature extraction options, metrics, and assumptions about speaker independence between the literature [1,2,12,13]. These problems render protocol conscious and reproducible analysis particularly significant in SER research.

Corpora like CREMA-D are still significant since they have controlled emotional labels and offer a large speaker diversity hence useful in the study of generalization across speakers [3]. But how such datasets are divided influences significantly the interpretation of results. Sampler-level splits can include samples of the same speaker in training and testing sets: this can also inflate performance by enabling models to use speaker-specific information or recording artifacts instead of emotion-specific signals [1,2]. By comparison, actor and leave-one-speaker-out are more stringent and realistic estimates of unseen-speaker deployment robustness [12,13,21]. Consequently, the current SER studies focus more on the significance of protocol-conscious reporting and transparent experimental design [12,13].

Traditional SER systems are still important since they are interpretable, computational efficient, and can be competitive when limited data is considered. These methods typically use manually designed acoustic features like MFCCs, pitchestatistics, energy-based features, and spectral features and then classifiers, including Support Vector Machines (SVMs) [4,5]. One-vs-rest SVM approaches are very common in multi-class scenarios as natural extensions of binary margin-based classification [14]. These models still remain powerful and reproducible baselines, but might be less effective at modeling the complex spectro-temporal patterns of emotion and could be worse at stringent speaker-independent tests [1,2]. Moreover, excitation-source-based modeling has also been demonstrated to reproduce emotionally relevant cues, which further supports the importance of a well-thought handcrafted representation in SER-related tasks [51,52].

Deep learning, particularly CNN-based modeling of log-mel spectrograms, has become common because convolutional filters can learn discriminative local spectro-temporal patterns directly from speech representations [6,7]. Robustness can be improved through augmentation and regularization methods such as SpecAugment-style masking, focal loss, and mixup, which encourage distributed feature use, emphasize difficult samples, and promote smoother decision boundaries [8–10]. Adam optimization, librosa-based preprocessing, and established digital signal-processing principles are also widely used in practical SER implementations [11,15,16]. Nevertheless, compact CNNs can remain sensitive to speaker mismatch and acoustically similar emotion classes, especially when datasets are limited or acted [1,13]. Transformer-style and attention-based models can capture longer-range dependencies and global emotional context [17–19], while ensemble and fusion methods are frequently investigated to improve stability across evaluation conditions [19,20].

Self-supervised learning (SSL) has recently become a significant reference point in speech and paralinguistic modeling. wav2vec 2.0, HuBERT, and WavLM are pretrained encoders with strong transferability to downstream speech tasks [46–48]. In affective and related paralinguistic applications, wav2vec 2.0 has been used for vocal emotion recognition and stuttering analysis, while pretrained wav2vec and HuBERT features have also shown potential in dysarthria classification [53,54]. The present study therefore does not claim to replace or outperform SSL-based state-of-the-art systems. Its contribution is a lower-compute, protocol-aware reference framework that isolates the effects of split strategy and representation fusion under one common pipeline. By reporting random and actor-wise results, five-seed variability, class-wise errors, and deployment using the same preprocessing, the study addresses an empirical reproducibility question that is distinct from proposing a new backbone. Direct benchmarking against SSL models is retained as future work.

## 3 Proposed method

### 3.1 Overall pipeline

This study implements a protocol-aware SER pipeline for fair comparison among three systems: (i) MFCC+SVM, (ii) a log-mel CNN, and (iii) a hybrid model that concatenates handcrafted features with CNN embeddings before final SVM classification. All systems share the same waveform standardization, class definitions, and evaluation metrics so that differences in performance can be attributed primarily to modeling choices rather than preprocessing mismatch [3,13,21].

As illustrated in Fig 2, each input waveform is converted into two synchronized representations. The handcrafted branch produces pooled utterance-level descriptors, while the deep branch produces a fixed-size log-mel spectrogram for CNN-based representation learning. The hybrid model then fuses the handcrafted vector with the penultimate-layer CNN embedding and performs final classification using an SVM.

The method intentionally uses established components so that the central experimental question can be isolated: whether complementary handcrafted and compact deep representations provide consistent gains when preprocessing, split construction, metrics, and repeated-run reporting are held constant. Therefore, the methodological contribution is the controlled design and validation of the integrated pipeline rather than a claim that MFCC, CNN, SVM, or feature concatenation is individually novel.

### 3.2 Preprocessing

All utterances are mono-sampled and resampled to a fixed sampling rate of *f*_*s*_. In order to maintain the consistent length of input, the waveform is center-cropped or zero-padded to a fixed length, which results in a sequence.


$$\begin{array}{c} x=\left\{x_{\text{1}},x_{\text{2}},\ldots ,x_{N}\right\}. \end{array}$$ (1)

This fixed-duration policy reduces variability arising from leading/trailing silence and recording-length differences, while also enabling efficient batch processing in deep models. Such normalization is particularly important in CNN-based SER, where convolutional feature learning can be sensitive to input alignment and temporal scale [20]. In speech-processing pipelines, center cropping is commonly applied to maintain uniform time-frequency resolution and stable batching in neural networks, while reducing bias toward early silence segments in an utterance. Resampling and fixed-length normalization are likewise standard practices for achieving consistent input representations [25]. Furthermore, fixed-duration inputs help reduce variability in spectrogram statistics caused by silence and recording length, which can affect the behavior of downstream classifiers [26].

### 3.3 Feature extraction

#### 3.3.1 Handcrafted branch: MFCC statistics.

Features of handcrafted computation are calculated based on Mel-Frequency Cepstral Coefficients (MFCCs), which is one of the established families of speech descriptors and has been extensively relied on the traditional SER baselines [4]. MFCCs are obtained at short-time frames, and the result is a coefficient matrix, denoted as $C\in R^{T\times K}$where *T* is the amount of frames and *K* is the amount of coefficients. Statistics are calculated to obtain a fixed length description of each coefficient dimension *k*:


$$\begin{array}{c} {\;\mu }_{k}=\frac{\text{1}}{T}\sum_{t=\text{1}}^{T}C_{t,k},\;\;\sigma_{k}=\sqrt{\frac{\text{1}}{T}\sum_{t=\text{1}}^{T}{\left(C_{t,k}-\mu_{k}\right)}^{\text{2}}} \end{array}$$ (2)

The final handcrafted vector is denoted $f_{\text{MFCC}}\in R^{d_{h}}$. Statistical pooling is employed due to the fact that it gives a concise summary of all the data and is efficient to classical classifiers but also captures global distributional clues like spectral shape and affect-related articulation differences. These descriptors also provide an interpretable baseline for comparing representation learning approaches [21]. Beyond MFCCs, prior SER work frequently uses curated paralin- guistic feature sets that include energy, spectral, and voice-quality descriptors designed for affective speech analysis [24]. Such handcrafted descriptors are still widely used as strong baselines because they are computationally light and provide interpretable cues linked to prosody and voice quality [26].

#### 3.3.2 Deep branch: Log-mel spectrogram.

Log-mel spectrograms of the Short-Time Fourier Transform (STFT), a common time-frequency representation in speech processing, are used as deep models in this study [16]. The raw speech signal is converted first to a time frequency representation and then a power spectrogram is derived. An mel filterbank is then used to map the spectral data to mel-scaled frequency bands, which are then logarithmically compressed to make the log-mel spectrogram. The optional per-feature normalization (e.g., z-score normalization) can be used to stabilize the distribution of the features prior to model training as well. This gives the resulting normalized log-mel representation which is center-cropped or padded to a constant number of time frames and fed into the CNN. The use of log-mel features Log-mel features are popular in current SER since they maintain a perceptually significant frequency resolution, and offer a consistent and useful input representation to convolutional learning [8,20].

### 3.4 Models

#### 3.4.1 Baseline 1: MFCC + SVM.

Support Vector Machines (SVMs) are a strong classical baseline for high-dimensional features and remain widely used in speech classification and benchmarking due to their robustness and simplicity [5]. Given handcrafted features *f*_MFCC_, a linear SVM learns a margin-maximizing separator by solving:


$$\begin{array}{c} \underset{w,b,\xi }{\text{min}}\;\frac{\text{1}}{\text{2}}∥w{∥}^{\text{2}}+C\sum_{i}\xi_{i}\;\text{s}.\text{t}.\;y_{i}\left(w^{⊤}x_{i}+b\right)\geq \text{1}-\xi_{i},\;\xi_{i}\geq \text{0}, \end{array}$$ (6)

where *C* controls the trade-off between margin and misclassification. For multi-class SER, a one-vs-rest strategy is typically employed [14]. This baseline serves as an efficient reference point and is especially relevant under CPU constraints, where low-latency inference may be required for interactive deployment.

#### 3.4.2 Baseline 2: Log-mel CNN.

A convolutional neural network (CNN) is trained on log-mel spectrograms to learn discriminative time–frequency patterns associated with emotion. The classifier outputs $z\in R^{C}$and softmax probabilities:


$$\begin{array}{c} p\left(y=c|\tilde{S}\right)=\frac{\text{exp}\left(z_{c}\right)}{\sum_{i=\text{1}}^{C}\text{exp}\left(z_{i}\right)}. \end{array}$$ (7)

The standard multi-class cross-entropy is:


$$\begin{array}{c} L_{\text{CE}}=-\sum_{c=\text{1}}^{C}y_{c}\text{log}\left(p_{c}\right). \end{array}$$ (8)

CNN-based SER is motivated by the ability of convolutional filters to learn localized spectro-temporal patterns—such as harmonics, formant shifts, spectral energy variations, and temporal dynamics—that correlate with emotional expression [19,20]. However, these models can remain sensitive to speaker mismatch and confusable emotion pairs, which motivates the robust training strategies described next. CNNs operating on spectrograms were first popularized in broader audio-classification benchmarks and were subsequently adopted in SER because they can efficiently capture local spectro-temporal structure while remaining relatively lightweight for deployment [6,27]. Nevertheless, deep spectrogram-based models may still overfit dataset-specific speaker or channel cues when evaluated using random splits, reinforcing the importance of speaker-independent testing [28,29].

### 3.5 Robust training strategies

The CNN branch is augmented with SpecAugment-style time and frequency masking to improve robustness and reduce overfitting. The masking operation removes contiguous temporal regions or mel-frequency bands, encouraging invariance to local distortions and reducing dependence on narrow spectral cues [8]. Although originally proposed for automatic speech recognition, this perturbation is now widely used in speech representation-learning pipelines because of its simplicity and effectiveness [8,21]. Focal loss is also used to emphasize hard and confusable emotion classes, such as neutral versus sad or fear versus disgust, by down-weighting easy examples and assigning greater influence to difficult samples [9,13]. Mixup regularization further improves generalization by training on convex combinations of inputs and labels, which promotes smoother decision boundaries and reduces reliance on speaker-specific artifacts [10,21]. Model parameters are optimized with Adam, which adapts per-parameter learning rates using first- and second-moment estimates and is commonly used in deep speech pipelines because of its stable convergence [15].

### 3.6 Proposed hybrid fusion model

The proposed hybrid model combines complementary information from handcrafted descriptors and deep embeddings. After training the CNN, an embedding vector is extracted from the penultimate layer:


$$\begin{array}{c} f_{\text{CNN}}\in R^{d_{d}}. \end{array}$$ (11)

This is fused with handcrafted features using concatenation:


$$\begin{array}{c} f_{\text{hyb}}=f_{\text{MFCC}}⊕f_{\text{CNN}}\in {\mathbb{R}}^{d_{h}+d_{d}}. \end{array}$$ (12)

An SVM is then trained on the fused vector to perform final classification. Handcrafted statistics capture stable utterance-level spectral and prosodic properties, whereas CNN embeddings encode learned time-frequency patterns; their combination can therefore reduce failure cases of either representation alone [4,5,20]. The fusion operation is deliberately simple and should not be interpreted as a new mathematical fusion mechanism. Its value in this study is as a controlled, low-compute integration point for testing whether complementary representations yield consistent gains under both speaker-overlapping and speaker-independent conditions. The design is also suitable for CPU deployment because the compact CNN functions as a feature extractor while final classification is handled by a lightweight SVM.

### 3.7 Evaluation protocol and reporting

All models are evaluated under the same random and actor-wise protocols and reported using Accuracy, UAR, Macro-F1, and confusion matrices. Results are aggregated across five random seeds as mean ± std to reduce dependence on a single split. In addition, Fig 2 summarizes the complete experimental flow from standardized input processing to protocol-aware evaluation.

## 4 Experimental setup

### 4.1 Dataset

All models are evaluated on the CREMA-D speech emotion dataset, which contains 7,442 utterances spoken by 91 actors and annotated into six discrete emotions: angry, disgust, fear, happy, neutral, and sad [3]. CREMA-D is frequently used in SER because it provides high speaker diversity and standardized categorical labels, enabling controlled within-dataset recognition experiments as well as speaker-independent generalization analysis. All recordings are processed as mono waveforms, and labels are maintained in an identical six-class space across all methods, so that performance differences arise from modeling decisions rather than label-processing variations.

### 4.2 Preprocessing and input standardization

Each of the audio files is subjected to a single preprocessing pipeline to create a fairness be- tween approaches. The signals are initially converted to mono and resampled to a fixed rate of sampling frequency *f*_*s*_. Resampling is a standard requirement for reproducible time–frequency analysis because STFT and mel-filterbank resolution depend on sampling rate [25]. The next step is to apply a fixed period to any utterance: longer utterances are center-cropped and shorter utterances are zero-padded. This produces equal-length waveforms, reduces variability caused by differing recording durations and leading/trailing silence, and guarantees consistent tensor shapes for CNN training and evaluation.

To reduce sensitivity to amplitude differences and channel variability, we apply consistent normalization choices at the feature stage (Section 4.3). This “same-input-policy” ensures that both handcrafted features (MFCC statistics) and spectrogram inputs are derived from the iden- tical standardized waveform, preventing confounds where one model benefits from a different preprocessing pipeline.

### 4.3 Feature representations

**Handcrafted branch (MFCC statistics):** For the MFCC+SVM baseline and the handcrafted component of the hybrid model, MFCCs are computed over short-time frames as standard speech descriptors [4]. To obtain a fixed-dimensional utterance representation, frame-wise MFCC sequences are summarized via statistical pooling (mean and standard deviation per co- efficient), yielding compact features suitable for efficient SVM learning and CPU inference. In addition to MFCCs, prior paralinguistics literature frequently highlights curated acoustic descriptors (energy, spectral, and voice-quality cues) as strong interpretable baselines for af- fective speech analysis, motivating the use of pooled utterance-level statistics in classical SER comparisons [24,26].

**Deep branch (log-mel spectrogram):** For deep models, waveforms are transformed into log-mel spectrograms using STFT, mel filter banks, and logarithmic compression. The mel scale is widely used in audio modeling because it provides perceptually motivated frequency spacing and works effectively with convolutional filters [30,31]. To reduce amplitude/channel variability, per-feature normalization (z-score) is applied. Finally, the spectrogram is center- cropped/padded to a fixed number of frames so that the CNN receives inputs with identical dimensions.

### 4.4 Evaluation protocols

To obtain a reliable and protocol-aware assessment, all models are evaluated under two complementary split strategies. Both splits use the same preprocessing and feature extraction settings (Table 1) so that performance differences reflect protocol strictness and model design rather than configuration changes. Following common reproducibility guidance, random-split (potentially speaker-overlapping) performance is clearly separated from speaker-independent performance [13,21].

**Table 1:** Key hyperparameters used in preprocessing, feature extraction, and CNN training.

Component	Setting (Value)
Sampling rate	*f_s_* = 22050 Hz
Fixed waveform duration	5.0 s
Waveform offset	0.0 s
Mel bins	*n*_mels_ = 128
Max mel frequency	*f*_max_ = 8000 Hz
Fixed spectrogram length	*T* = 400 frames (center-crop/pad)
Spectrogram normalization	z-score: (*S* − *µ*)*/*(*σ* + *ϵ*)
Optimizer (CNN)	Adam [15]
Loss (CNN)	Cross-entropy / focal loss option [9]
Mixup	*α* = 0.2 (Beta(*α, α*)) [10]
Focal loss parameters	*γ* = 2.0, *α* = 0.25 [9]
SpecAugment masking	time mask + freq mask [8]

- **Random split:** Utterances are divided into training and testing partitions using stratification by emotion class where applicable. Random splits are common because they are simple and often yield strong performance numbers, but they can allow the same speaker to appear in both train and test sets, which may inflate performance through speaker leakage [13]. Therefore, random-split results are interpreted as an optimistic estimate of within-dataset performance.
- **Actor-wise (speaker-independent) split:** Speakers are strictly separated between training and test sets so that no speaker identity overlap occurs across partitions. This protocol prevents leakage and provides a more realistic estimate of generalization to unseen speakers [21]. Actor-wise splitting is implemented using group-based partitioning, ensuring that all utterances from a given actor remain in a single split, consistent with standard group-aware evaluation practices in ML [32].

Across both protocols, Accuracy, Unweighted Average Recall (UAR), and Macro-F1 are reported. These are standard SER metrics; UAR and Macro-F1 are particularly informative when emotions are confusable or class-wise separability is uneven [22,23]. In addition, confusion matrices (raw and normalized) are reported to analyze systematic confusions between emotion pairs that may be hidden by scalar metrics alone [22].

### 4.5 Repeated runs and reporting

To reduce sensitivity to a single partition or initialization, each experimental setting is repeated across five different random seeds and summarized using mean ± std. Multi-run reporting improves the reliability of empirical comparisons and helps distinguish consistent gains from seed-specific variance [21]. For each seed, confusion matrices are also generated to support qualitative error-pattern analysis.

### 4.6 Implementation and runtime setting

All experiments are implemented in Python for reproducibility and ease of deployment. Feature extraction is implemented using librosa [11]. Classical models (SVM) and splitting utili- ties are implemented using scikit-learn, which provides standardized implementations for supervised learning and evaluation [33]. The deep learning components are implemented using TensorFlow/Keras [34,35], and CNN training uses the Adam optimizer due to its stable convergence and adaptive learning-rate behavior [15].

All experiments are executed on **CPU-only** hardware (no GPU acceleration). This reflects realistic deployment constraints and aligns the experimental environment with the end- to-end Streamlit inference interface used for interactive testing on unseen.wav files [36]. CPU-oriented evaluation also highlights the practicality of efficient feature representations and lightweight model designs for real-world SER integration.

### 4.7 Robust training and regularization

CNN-based SER can degrade under speaker mismatch and confusable emotion categories. To improve generalization and mitigate overfitting, widely adopted robustness strategies are incorporated during CNN training:

- **SpecAugment-style masking:** Time and frequency masking are applied directly to log-mel spectrograms, encouraging invariance to local occlusions and reducing reliance on narrow cues [8].
- **Focal loss:** Focal loss down-weights easy samples and emphasizes harder examples, improving learning under systematic confusions [9].
- **Mixup:** Mixup trains on convex combinations of inputs and labels, smoothing decision boundaries and typically improving generalization [10].

Together, these strategies provide complementary benefits (invariance, hard-sample emphasis, and boundary smoothing) and are commonly recommended to improve reliability under distribution shift and to reduce overfitting to speaker overlap [13,21].

### 4.8 Hyperparameter disclosure

For reproducibility, the key hyperparameters used throughout the pipeline are explicitly reported, including sampling rate $f_{s}$, fixed crop duration (or fixed spectrogram frame length), mel-bin resolution $n_{\text{mels}}$, frequency range, and CNN training configuration (optimizer and robustness settings). These settings are kept identical for both random and actor-wise protocols so that performance differences primarily reflect protocol strictness and model design rather than tuning changes.

## 5 Evaluation metrics and reporting

To provide a consistent and interpretable comparison, performance is reported using three complementary measures: Accuracy, Unweighted Average Recall (UAR), and Macro-F1. Accuracy reflects overall correctness, but it may be overly optimistic when class distributions are uneven or when certain emotions dominate predictions. For this reason, Macro-F1 and UAR are also reported, as these metrics are commonly recommended for multi-class evaluation because they reduce sensitivity to class prevalence by averaging performance across classes [22,23].

UAR is computed as the mean of per-class recall values, assigning equal importance to each emotion class:

Macro-F1 is computed by first calculating the F1-score independently for each class and then averaging:

As part of good practice in classifier evaluation, confusion matrices are also used to visualize class-wise behavior, and they help reveal systematic confusions between emotion pairs that may not be identified from scalar metrics alone [22]. Both raw-count confusion matrices and normalized (%) versions are reported to support qualitative comparison across classes.

To reduce sensitivity to a single split or random initialization, all experiments are repeated over five random seeds and summarized as mean ± std. The aggregated results (Table 2) are used for the main comparison, while per-seed results and confusion-matrix summaries are provided for transparency and error-pattern analysis. In addition, SER often exhibits class imbalance and confusable classes, so reporting class-balanced metrics is recommended in imbalanced classification settings [37]. To complement point estimates, mean ± std is reported over multiple seeds, and statistical testing is encouraged where appropriate (e.g., paired comparisons across folds/splits), following common guidance for empirical ML evaluation [38,39].

**Table 2:** Mean±std performance (%) over 5 seeds on CREMA-D under random and actor-wise protocols.

Split	Model	Acc.	UAR	Macro-F1
	MFCC+SVM	56.00 ± 1.10	56.05 ± 1.14	55.72 ± 0.95
Random	Log-mel CNN	51.74 ± 1.31	52.09 ± 1.30	50.20 ± 1.51
	Hybrid (Proposed)	**62.16%** ± **0.99%**	**62.34%** ± **1.00%**	**62.03%** ± **0.94%**
	MFCC+SVM	52.55 ± 1.76	52.46 ± 1.76	52.05 ± 1.95
Actor-wise	Log-mel CNN	46.09 ± 1.50	46.47 ± 1.57	42.68 ± 1.84
	Hybrid (Proposed)	**58.27%** ± **1.21%**	**58.25%** ± **1.17%**	**58.09%** ± **1.36%**

## 6 Results and discussion

### 6.1 Overall performance (mean±std over 5 seeds)

Table 2 reports mean±std results over five random seeds for both evaluation protocols. Across all metrics (Accuracy, UAR, and Macro-F1), the proposed hybrid fusion model consistently achieves the best performance in both the random and actor-wise settings. This indicates that combining handcrafted statistics with learned CNN embeddings provides complementary in- formation that improves classification stability.

On the random split, the hybrid model reaches 62*.*16% ± 0*.*99% Accuracy and 62*.*03% ± 0*.*94% Macro-F1, outperforming MFCC+SVM (56*.*00% ± 1*.*10% Accuracy) and the log-mel CNN baseline (51*.*74% ± 1*.*31% Accuracy). On the actor-wise split, overall scores decrease for all models due to speaker mismatch, but the hybrid model remains strongest with 58*.*27% ± 1*.*21% Accuracy and 58*.*09% ± 1*.*36% Macro-F1.

This protocol gap is expected because speaker-independent testing is a stricter evaluation setting that reduces the benefit of speaker overlap present in random splits. UAR and Macro-F1 are reported in addition to Accuracy because SER often exhibits class confusability and uneven per-class separability, where aggregate Accuracy alone may not adequately reflect class-balanced behavior [22,23].

### 6.2 Confusion matrix analysis (per-class errors)

**Fig 3:**
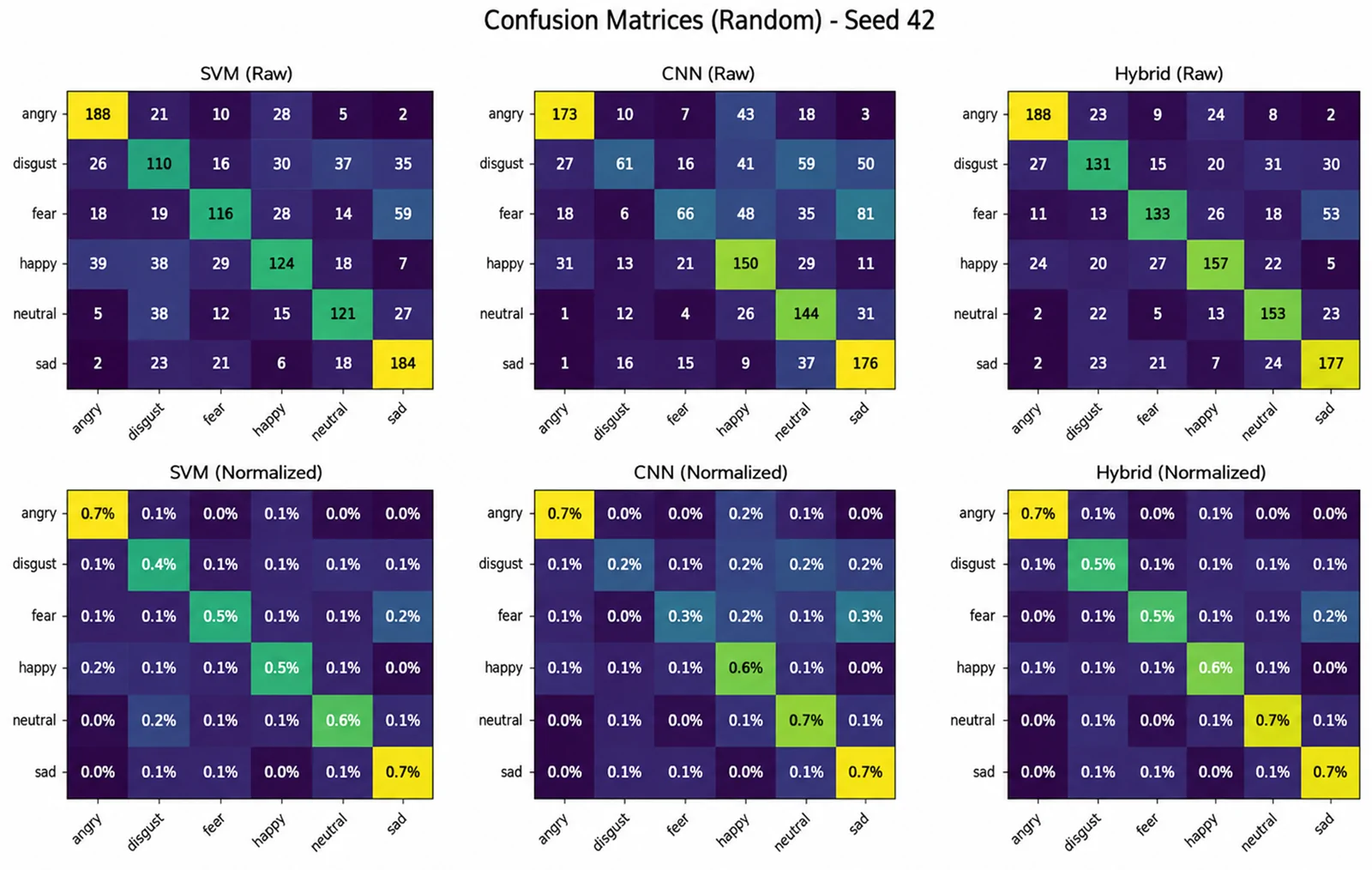
Random-split confusion matrices for SVM, CNN, and Hybrid using raw and normalized values.

**Fig 4:**
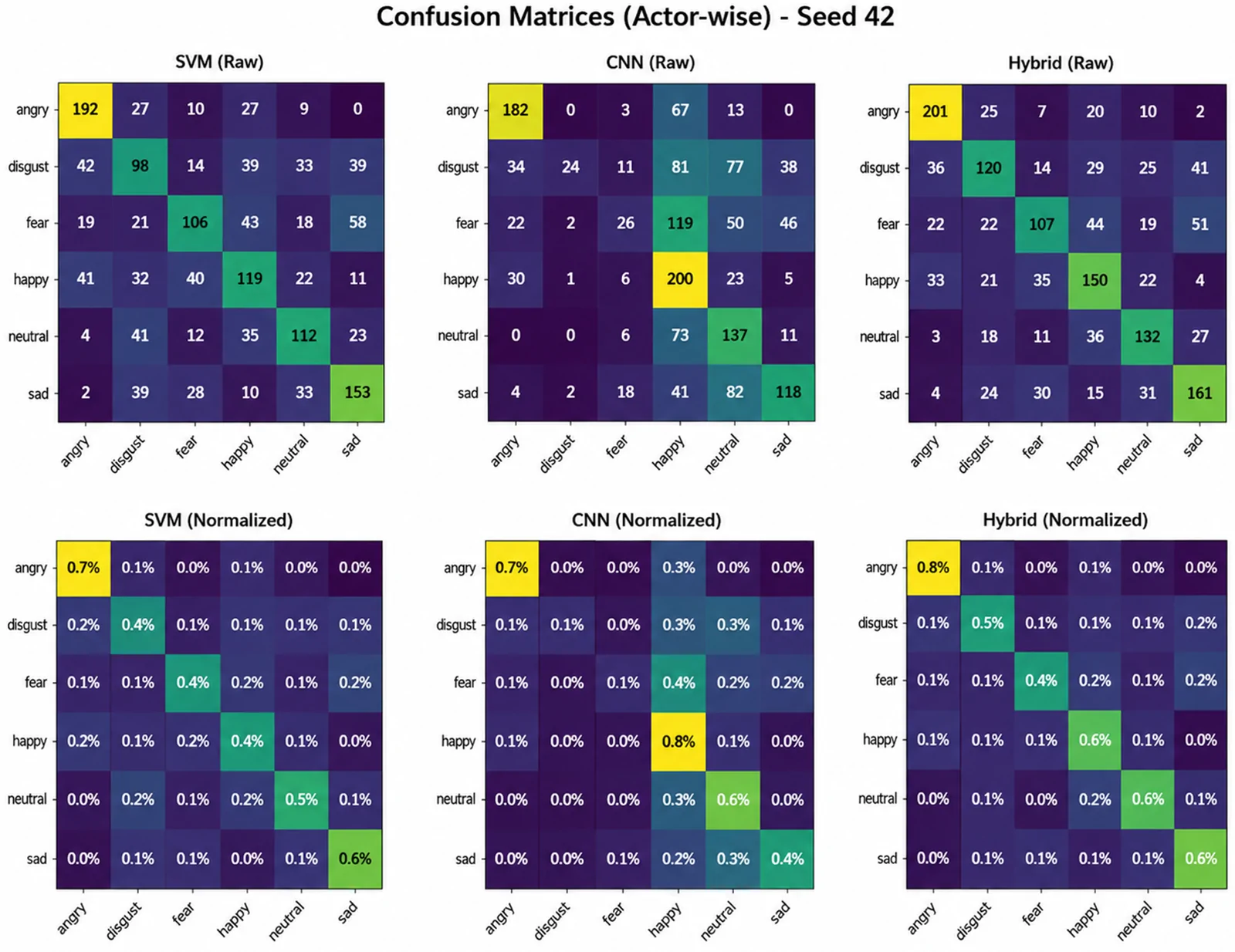
Actor-wise confusion matrices for SVM, CNN, and Hybrid using raw and normalized values.

To examine class-wise behavior beyond aggregate metrics, Figs 3 and 4 present representative confusion matrices (raw counts and normalized percentages) for the random and actor-wise protocols, respectively. Confusion matrices are included because they explicitly show which emotion pairs are systematically confused and whether error patterns become more pronounced under speaker-independent evaluation. This form of per-class analysis complements Accuracy by revealing imbalance-like behavior and asymmetric confusions that are common in multi-class recognition tasks [22,23].

Overall, stronger diagonal dominance is observed in the random split, whereas the actor-wise split exhibits increased off-diagonal mass, indicating reduced generalization to unseen speakers. Across models, the hybrid system generally produces cleaner diagonals and fewer confusions than the individual baselines, which is consistent with the performance improvements reported in Table 2. Confusion matrices are widely used in diagnostic evaluation because they expose per-class error structure and systematic confusions that may be hidden by aggregate scores alone [40].

### 6.3 Comparison of SVM vs CNN vs hybrid

The three evaluated systems represent complementary modeling choices. MFCC+SVM pro- vides a strong, computationally light baseline that benefits from stable utterance-level statis- tics, and it remains competitive under CPU-only inference. The log-mel CNN learns time– frequency patterns directly from spectrogram structure, but its performance degrades more strongly under speaker-independent testing, suggesting higher sensitivity to speaker/channel variation and to limited training data. This behavior aligns with the protocol gap seen between the random and actor-wise settings in Table 2.

The hybrid fusion model consistently performs best because it combines two complementary information sources: (i) handcrafted descriptors that summarize stable spectral and prosodic statistics, and (ii) CNN embeddings that capture discriminative high-level time-frequency cues. Relative to MFCC+SVM, the hybrid model improves Accuracy by 6.16 percentage points on the random split (62.16 vs. 56.00) and 5.72 points on the actor-wise split (58.27 vs. 52.55). Relative to the log-mel CNN baseline, the gains are 10.42 points and 12.18 points, respectively. These results do not establish universal algorithmic superiority; rather, they show that within the controlled CREMA-D protocol, the integrated representation is more stable than either branch alone and that the advantage persists when speakers are separated between training and testing.

The confusion matrices (Figs 3–4) further support this conclusion. Compared to the stan- dalone baselines, the hybrid system generally shows a stronger diagonal and reduced off- diagonal spillover, especially under actor-wise testing, which is the more realistic deployment scenario. Therefore, the proposed fusion design provides a practical accuracy/robustness trade-off while remaining compatible with CPU-based real-time inference.

### 6.4 Deployment and practical application (streamlit)

To improve usability and practical reproducibility, an optional Streamlit-based deployment is developed to provide both (i) access to research outputs and (ii) live inference through a simple web interface. The application offers a unified dashboard for comparing SVM, CNN, and the proposed Hybrid model under both random and actor-wise protocols, while also supporting downloads of the exact outputs used in the study (CSV tables and figures) and testing on new WAV inputs. Such qualitative inspection is important in affect recognition because emotion classes can overlap acoustically, and confusion patterns often reveal model biases (e.g., systematic neutral–sad confusions) more clearly than any single scalar metric [40].

The interface is organized into a model-status panel indicating available model/split artifacts, a results section summarizing 5-seed mean ± std performance along with per-seed metrics, a confusion-matrix viewer for inspecting raw-count and normalized (%) matrices with download options, and a dedicated WAV-upload section for live prediction on unseen audio. The application also supports direct export of outputs for thesis writing and reproducible analysis, including aggregated performance results (mean ± std) in CSV format, per-seed evaluation metrics in CSV format, confusion-matrix summary figures for random and actor-wise protocols in PNG format, and bulk confusion-matrix data (e.g., zipped images or saved numeric arrays) for downstream analysis and reproducible visualization.

For live prediction, a user uploads a WAV file and the application applies the same preprocessing pipeline used during experimentation (e.g., fixed sampling rate and feature configuration), then returns a predicted emotion label and confidence scores. This enables demonstration and hands-on testing on unseen samples without command-line execution.

The deployment is designed to reduce train–test mismatch and preserve repeatable outputs by reusing the same preprocessing and feature-extraction settings applied during training, loading saved model checkpoints and stored evaluation artifacts (including seed-wise results and confusion matrices), and providing downloadable outputs in standard formats (CSV/PNG/arrays) for independent verification and thesis integration. The browser-based interface also improves accessibility by allowing non-technical users to inspect evaluation results and run live predictions without code modification. Overall, this deployment layer serves as a bridge between research and implementation, improving accessibility while preserving a reproducible experimental workflow.

## 7 Conclusion

This study presents a protocol-aware comparison of classical, deep-learning, and hybrid speech emotion recognition pipelines on CREMA-D under both random and actor-wise (speaker-independent) evaluation. Across Accuracy, UAR, and Macro-F1, the hybrid fusion model achieves the strongest mean performance over five seeds. The decline observed for every model under actor-wise testing confirms that speaker-independent evaluation is more demanding and provides a more realistic estimate of deployment performance than a potentially speaker-overlapping random split.

The contribution is methodological and practical rather than the proposal of a new fundamental classifier. By holding preprocessing and reporting conditions constant, the study shows that combining handcrafted utterance-level descriptors with compact CNN embeddings provides consistent gains over either representation alone. The side-by-side protocol comparison, multi-seed uncertainty reporting, class-wise error analysis, and CPU-oriented Streamlit implementation together provide a transparent reference workflow for lightweight and reproducible SER experimentation.

## 8 Limitations and future work

Because the individual components are established, this work should be interpreted as a reproducible lightweight benchmark and integration study rather than a new state-of-the-art architecture. The empirical findings are limited to CREMA-D and within-dataset evaluation, and the current experiments do not include direct comparison with modern self-supervised speech encoders. Future work will therefore extend the benchmark to RAVDESS [41], IEMOCAP [28], EMO-DB [42], and MSP-IMPROV [43], including cross-dataset testing to quantify distribution shift; compare the lightweight hybrid pipeline directly with wav2vec 2.0, HuBERT, and WavLM [46–48,53,54]; and examine noise-aware augmentation, domain adaptation, calibration, and statistical significance testing so that performance and confidence estimates are more reliable for downstream use [44,45,49,50].

## Supporting information

S1 File Experimental Results* for dataset1. (CSV)

S2 File Cross-Validation Results* for dataset2. (CSV)

S3 File Partial CREMA-D Speech-Emotion Dataset. ZIP* for partial_dataset. (ZIP)
